# Nutritional and Sensory Properties of Protein Hydrolysates Based on Salmon (*Salmo salar*), Mackerel (*Scomber scombrus*), and Herring (*Clupea harengus*) Heads and Backbones

**DOI:** 10.3389/fnut.2021.695151

**Published:** 2021-12-10

**Authors:** Tone Aspevik, Silje Steinsholm, Birthe Vang, Mats Carlehög, Jan Arne Arnesen, Katerina Kousoulaki

**Affiliations:** ^1^Department Nutrition and Feed Technology, Nofima, Fyllingsdalen, Norway; ^2^Department Marine Biotechnology, Nofima, Tromsø, Norway; ^3^Department Consumer and Sensory Sciences, Nofima, Ås, Norway

**Keywords:** salmon (*Salmo salar*), mackerel (*Scomber scombrus*), herring (*Clupea harengus*), side-stream materials, protein hydrolysis, sensory properties

## Abstract

Protein hydrolysates based on salmon, mackerel, and herring heads and backbones were produced, and the sensory properties of the hydrolysates were evaluated by a highly trained sensory panel. The nutritional content of the products was evaluated, and the hydrolysates contained all the amino acids inherent to the raw material, including considerable levels of connective tissue amino acids glycine, proline, and hydroxyproline. Hydrolysates based on herring were the most flavor intense, whereas hydrolysates based on salmon were deemed more palatable. In this work, choice of fraction (heads vs. backbones) and enzyme had minor effects on sensory and nutritional properties, indicating that choice of raw material species was the major factor for flavor development in the produced protein hydrolysates. There were large variations in protein content and amino acid composition in the raw material fractions, but as expected, only minor variations were found in the final products.

## Introduction

Side-stream materials from fishing and aquaculture, such as heads, backbones, viscera, and trimmings may comprise up to 70% of the whole fish (1). In Norway, all side-stream materials obtained from salmon aquaculture, with the exception of blood, are utilized. The side streams are used for both feed and food products. Side stream materials from mackerel and herring are mainly used for lower-value feed ingredients (2). The Norwegian pelagic fisheries volumes in Norway have been rather stable the last decade, amounting to over 1.3 million tons. In 2019, 560,000 tons herring and ~159,000 tons mackerel were landed (3). Most of the herring from Norwegian fisheries are fileted, whereas mackerel is mostly sold as round frozen (2). However, the growing share of mackerel that is fileted is generating increasing amounts of heads and backbones available for valorization [194,000 tons in 2019) (2)]. The raw materials have food grade quality after the primary processing and are suitable as food ingredients when handled correctly (4, 5).

The production of protein hydrolysates using commercial proteases is a promising approach for utilizing marine side stream materials. Enzymatic protein hydrolysis is a mild process that facilitates increased recovery of water-soluble peptides and oil from the raw material (6, 7). Protein hydrolysates based on marine sources are rich in essential amino acids, making such products suitable as food additives or nutritional supplements (8). Furthermore, the peptides in salmon, mackerel, and herring protein hydrolysates may have antioxidative, antimicrobial and/or antihypertensive activities (8, 9). Provided that the raw material can be converted to a product suitable for inclusion in foods, the additional processing costs may be justified, both regarding increased raw material valorization and environmental sustainability.

Off-flavors in the final product remain a challenge in the production of enzymatic protein hydrolysates from (10–14). Water-soluble molecules present in the raw material will follow the aqueous hydrolysate phase and influence the product sensory properties. These components include inorganic salts, trimethylamine, nucleotides, protein and non-protein amino acids, and possibly small amounts of lipid oxidation products (14–16). Bitter taste, which is ascribed to small hydrophobic peptides (11, 17), is one of the main off-flavors found as challenges in protein hydrolysates for human consumption. However, the choice of enzyme and processing conditions may significantly influence the bitter taste development in the final protein hydrolysate (14, 18–20). The enzymes Food Pro PNL and Bromelain were selected based on previous experience. Food Pro PNL have proven to be cost effective (21) and provide low bitterness (14) in protein hydrolysates. Bromelain has a broad specificity and efficient on connective tissue (22). The enzymes were also chosen because their optimal pH conditions and are in line with the natural pH of the raw material allowing a process upscaling to be done without laborious and cost-enhancing pH adjustments.

Several works have addressed the taste and flavor development of marine protein hydrolysates (10, 11, 13–15, 18, 19, 23). Quality assessment of food grade hydrolysates must include evaluation of sensory properties, preferably performed by a trained taste panel (24). Descriptive analysis is a comprehensive method which includes training of the panelists to quantify specific sensory attributes for appearance, flavor, texture, and aftertaste. The assessors are calibrated on samples that are considered the most different on the selected attributes typical for the samples to be tested. However, such study-to-study calibrations makes interstudy comparisons difficult. Thus, sensory evaluation of hydrolysates based on different raw materials, at otherwise similar processing conditions, are necessary for the assessment of products destined for human consumption. In this work, enzymatic protein hydrolysates from salmon, mackerel, and herring heads and backbones were produced using similar hydrolytic conditions. The aim was to evaluate their proximate and nutritional composition, and sensory properties to compare their suitability for inclusion in food formulations.

## Materials and Methods

### Materials

Fresh farmed salmon (*Salmo salar*) was provided by Sotra Seafood, Øygarden, Norway. Frozen herring (*Clupea harengus*) and mackerel (*Scomber scombrus*) were provided by Pelagia, Norway. The raw materials were fileted, the heads and backbones were collected and ground using a kitchen grinder and kept frozen (−20°C) until hydrolysis. The proteases used were Bromelain BR1200 (EC 3.4.22.32, Enzybel, Waterloo, Belgium) and FoodPro PNL (EC 3.4.24.28, DuPont, Wilmington, DE). Peptide standards for analysis of molecular weight distribution were purchased from Sigma-Aldrich (Oslo, Norway) except lysozyme (Fluka biochemicals, Buchs, Switzerland) and Alberta standards (Alberta Peptide Institute, Department of Biochemistry, University of Alberta, Edmonton, Canada). All chemicals for analyses were of analytical grade.

### Methods

#### Chemical Methods

Nitrogen was analyzed by the Kjeldahl method (25) and crude protein estimated based on N × 6.25. Ash was determined by combustion at 550°C (26). Dry matter was determined by drying at 103°C (27). Molecular weight distribution was measured by HPLC size exclusion chromatography (SEC) (1260 series HPLC Agilent Technologies) using a Superdex Peptide 10/300GL column (GE Healthcare, Uppsala, Sweden), acetonitrile with TFA as eluent, and UV detection at 190–600 nm (14). Total amino acid composition was quantified by HPLC with fluorescence detection with excitation/emission at 250/395 nm. The proteins were hydrolyzed to free amino acids with 6N HCl, and amino acids were derivatized with 6-aminoquinolyl-*N*-hydroxysuccinimidyl carbamate before HPLC analysis (Waters Accq Tag 3.9 × 150 mm) and detector (28). The lipid content was analyzed by the EU 152/2009 (29) method with acid hydrolysis. Fatty acid analyses were carried out by AOCS Official Method (Ce 1b-89) (30). All analyses were done in duplicate measurements.

#### Enzymatic Hydrolysis

In all experiments, enzymes were added at similar activity of 10 U/g protein, based on a casein-activity assay (14). Hydrolyses were conducted in a R10 Bear Varimixer (A/S Wodschow & Co. Brøndby, Denmark) or Distek 7100 Bathless Dissolution Tester (Distek Inc. North Brunswick, NJ). An equal mass of tap water was added to the ground raw material. The mixture was heated to 50°C and then the enzyme was added. Stirring was continued at 50°C for 60 min. The temperature was raised to 90°C and held at that temperature for 10 min (to inactivate the proteases). The mixture was centrifuged at 15,000 g for 10 min (Sorvall LYNX 6000, Thermo scientific, Waltham, MA). The liquid was decanted from the sediment into a separatory funnel and the aqueous and oil were separated. The aqueous phase was clarified by cross flow ultrafiltration through a membrane with nominal molecular weight cut-off of 100 kDa (Vivaflow 200, Sartorius, Goettingen, Germany). The ultrafiltration permeate was freeze dried and stored at −30°C until sensory assessment.

#### Sensory Analysis

The freeze-dried hydrolysates were dissolved in tap water at a concentration of 1.0 wt % before sensory analysis. A highly trained panel of 10 assessors (10 women; aged, 37–64 years) performed a sensory descriptive analysis according to the “Generic Descriptive Analysis,” as described by Lawless and Heymann (24) and the ISO standard 13299 (31). The assessors were regularly tested and trained according to ISO standard 8586 (32), and the sensory laboratory followed the practice of ISO standard 8589 (33). The assessors agreed upon 17 attributes describing the hydrolysate samples (Table 1). Samples were served in glasses of plastic (20 ml) with a lid at a room temperature of 18°C ± 2°C. All attributes were evaluated on an unstructured 15 cm line scale with labeled end points going from “no intensity” (1) to “high intensity” (9). Each assessor evaluated all samples at individual speed on a computer system for direct recording of data (EyeQuestion, Software Logic8 BV, Utrecht, the Netherlands).

**Table 1:** The sensory attributes used in the sensory evaluation of salmon, mackerel, and herring protein hydrolysates.

**Attribute**	**Description**
Total intensity of flavor	The intensity of all tastes and flavors
Sweet taste	Basic sweet taste
Salty taste	Basic salt taste (sodium chloride)
Acidic taste	Basic acidic taste
Bitter taste	Basic bitter taste
Umami taste	Basic umami taste
Fish flavor	Flavor of boiled white fish
Marine flavor	The flavor of fresh, salty sea
Shellfish flavor	The flavor of shellfish, a sweet teste of shrimp, crab, and crayfish
Burnt flavor	Related to a burnt flavor
Rancid flavor	All rancid flavors (grass, hay, stearin, paint)
Fermented fish flavor	Related to flavors that remind of a pier, bad and stale fish
Flavorless flavor	Related to water from boiling of potatoes
Cloying flavor	Related to a sickening flavor
Astringency	A complex feeling of contraction and dryness of the mouth
Fatness	The fattiness of the products
Aftertaste	The intensity of the tastes and flavors left in the mouth after 30 s

In a pretest session before the main test, the assessors were calibrated on samples that were considered the most different on the selected attributes typical for the hydrolysate samples to be tested. All samples were served to the panel coded with a three-digit number in a balanced block design. Tap water and unsalted crackers were available for palate cleansing, and red light was used in the sensory laboratory to masque differences in appearance between samples.

#### Statistical Analysis

Analysis of variance (ANOVA) of the sensory profiling data was performed using Minitab (v19.2, Pennsylvania State University, PA). First, a two-way mixed effects ANOVA model was conducted to assess differences between products for all sensory attributes. Product was set as a fixed variable, whereas assessor and interaction effects were set as random variables (34). Mixed effects ANOVA was used to evaluate the individual fixed effects of specie, fraction, and enzyme on sensory attributes, still treating assessor as a random variable. Tukey's pairwise comparison was applied where significant (*p* < 0.05) differences were found. Principal component analysis (PCA) was performed using Unscrambler v.10.4.1 (Camo, Oslo, Norway) to evaluate the association between sensory properties and molecular weight distribution of the hydrolysates. Prior to analysis, all variables were mean centered and standardized.

## Results and Discussion

### Proximate Composition, Peptide Size Distribution, and Amino Acid Composition

All the raw materials contained high levels of protein and lipids (Table 2). The backbones contained most proteins and indispensable amino acids (IAA) relative to the heads for all species. As expected, all raw materials had high levels of the valuable polyunsaturated fatty acids (PUFAs). The salmon raw materials had much higher levels of *n*-6 PUFA than mackerel and herring, reflecting the amounts of plant oils used in salmon feed (35). All raw materials also contained high levels of the marine *n*-3 PUFA, EPA, and DHA, which are very susceptible to lipid oxidation and can influence the sensory properties of the final product (36). The proximate compositions were in agreement with other studies (9, 10), except the mackerel heads, which have slightly less ash content than previously reported (10), mainly ascribed to different batches of raw material and fileting methods used. The latter may give varying ratios of muscle tissue and bone, causing a displacement in the level of ash, which is mostly derived from the bone tissue.

**Table 2:** Proximate, lipid, and amino acid composition (g/100 g sample) of freeze-dried heads and backbones from salmon, mackerel, and herring^a^.

	**Salmon**		**Mackerel**		**Herring**	
	**Backbone**	**Head**	**Backbone**	**Head**	**Backbone**	**Head**
Protein	46.4	34.6	46.6	34.6	51.1	38.5
Lipids	48.6	54.4	42.9	54.4	43.6	42.8
Ash	8.1	9.1	8.5	9.1	6.9	16.9
Dry matter	99.1	98.1	98.0	98.1	98.5	97.7
Fatty acids^b^						
Saturated FA	13.8	20.6	20.6	20.6	18.3	17.5
MUFA	51.4	37.6	38.8	37.6	48.1	51.7
PUFA	29.3	18.7	20.0	18.7	21.2	14.2
PUFA n-6	13.7	13.8	2.0	2.0	1.7	1.5
PUFA n-3	15.9	16.4	13.7	16.3	19.1	12.4
EPA	2.7	4.6	4.9	4.6	6.5	3.9
DHA	5.1	6.7	6.9	6.7	7.2	4.8
Amino acids						
Arginine	3.0	2.0	2.9	2.0	2.9	2.4
Histidine	2.1	0.7	1.2	0.7	1.5	0.8
Isoleucine	2.0	1.0	1.7	1.0	2.1	1.2
Leucine	3.3	1.8	2.9	1.8	3.7	2.2
Lysine	3.7	2.2	3.6	2.2	4.4	2.2
Methionine	1.3	0.9	1.4	0.9	1.6	1.2
Phenylalanine	1.8	0.9	1.5	0.9	1.8	1.3
Threonine	2.0	1.2	1.8	1.2	2.0	1.3
Valine	2.4	1.2	2.1	1.2	2.6	1.6
Sum IAA^c^	21.6	11.9	19.1	11.9	22.6	14.4
Alanine	2.8	2.0	2.9	2.0	3.0	2.4
Aspartic acid	4.2	2.7	4.1	2.7	4.6	2.8
Glutamic acid	6.0	3.9	6.0	3.9	6.6	4.2
Glycine	3.8	3.7	4.5	3.7	3.0	4.2
Hydroxyproline	0.7	0.9	0.9	0.9	0.3	1.1
Proline	2.2	1.9	2.3	1.9	1.9	2.3
Serine	2.1	1.4	2.0	1.4	2.0	1.7
Tyrosine	1.2	0.9	1.5	0.9	1.7	1.1
Sum DAA^d^	23.0	17.4	24.2	17.4	23.1	19.7
Sum amino acids	43.3	31.2	44.6	31.2	45.7	34.1

*^a^Allowed replicate variation: Protein: ≤ 0.3%, Lipids: ≤ 0.54%, Ash: ≤ 0.3%, Dry matter: ≤ 0.2%, Fatty acids: ≤ 5% of mean of dominating acids, Amino acids: RSD <6% for 2/3 of amino acids.*

*^b^Lipid composition based on fat extracted from freeze dried raw material. FA: fatty acids, EPA: eicosapentaenoic acid, DHA: docosahexaenoic acid.*

*^c^Indispensable amino acids.*

*^d^Dispensable amino acids*.

All hydrolysates were high in protein and ash (Table 3) and especially, the head-based hydrolysates were richer in ash and lipids compared with the backbone-hydrolysates, in agreement with previous findings for salmon, mackerel (10), and herring (9). The difference in raw material proximate compositions did not affect the hydrolysate composition to a greater extent. Only minor variations in protein content could be found in the final dried products. This is as expected because the hydrolysate product only consisted of the filtrated water-soluble fraction containing mostly proteins, causing a displacement in composition. The sum of total amino acids is the most accurate estimate for protein content in a product (37), and the levels of total amino acids (Table 4) were slightly lower compared to that of analyzed total proteins (N × 6.25) (Table 3) for all hydrolysates. This provides evidence that the commonly used nitrogen-to-protein factor of 6.25 is inaccurate for the different fish fractions and substrates, and has been found to be closer to 5.2, and is of importance for the production of comparable hydrolysates (14). The hydrolysates contained all amino acids present in the raw material, with noticeable levels of glycine, proline, and hydroxyproline, predominant in fish connective tissue. This is in accordance with Liaset and Espe (38), who reported higher levels of these amino acids in side-stream based hydrolysates compared with filets. No apparent effect of the used enzyme on amino acid distribution was observed, suggesting similar release of proteins from the raw material. Hydrolysates based on backbones had slightly higher levels of IAA for all three species tested. The differences were, however, small and all hydrolysates were considered of high nutritional protein quality, based on daily requirements for adults. On average, an adult of 70 kg requires about 6 g of IAA per day (39), and all hydrolysates contained >30 g IAA/100 g dried hydrolysate.

**Table 3:** Proximate composition (g/100 g) of protein hydrolysates based on salmon (S), mackerel (M), and herring (H) backbones (B) and heads (H) using enzymes Food Pro (-FP) and Bromelain (-B)^a^.

	**Salmon**				**Mackerel**				**Herring**			
	**Backbone**		**Head**		**Backbone**		**Head**		**Backbone**		**Head**	
	**SB-FP**	**SB-B**	**SH-FP**	**SH-B**	**MB-FP**	**MB-B**	**MH-FP**	**MH-B**	**HB-FP**	**HB-B**	**HH-FP**	**HH-B**
Protein	92.1	91.4	92.3	89.3	89.9	91.6	88.7	88.3	84.9	87.8	82.6	83.0
Dry matter	97.0	95.0	94.2	94.9	95.9	97.0	97.5	96.4	96.0	95.7	96.1	95.7
Ash	8.0	7.2	11.5	10.8	9.5	8.4	11.6	10.7	9.6	8.9	11.4	11.4

*^a^Allowed replicate variation: Protein: ≤ 0.3%, Ash: ≤ 0.3%, Dry matter: ≤ 0.2%*.

**Table 4:** Amino acid composition (g/100 g sample)^a^ of protein hydrolysates based on salmon (S), mackerel (M) and herring (H) backbones (B) and heads (H), using enzymes Food Pro (-FP) and Bromelain (-B).

	**Salmon**				**Mackerel**				**Herring**			
	**Backbone**		**Head**		**Backbone**		**Head**		**Backbone**		**Head**	
	**SB-FP**	**SB-B**	**SH-FP**	**SH-B**	**MB-FP**	**MB-B**	**MH-FP**	**MH-B**	**HB-FP**	**HB-B**	**HH-FP**	**HH-B**
IAA^2^												
Arginine	6.6	6.7	6.7	6.5	5.3	5.2	5.3	5.6	4.5	5.0	4.8	5.0
Histidine	2.2	2.2	1.9	1.8	3.8	3.8	2.7	2.6	2.0	2.0	1.5	1.5
Isoleucine	2.5	2.6	2.1	2.0	2.4	2.6	2.5	2.5	2.6	2.6	2.4	2.2
Leucine	5.1	5.3	4.2	4.2	4.9	5.2	5.0	5.1	5.6	5.6	5.1	5.0
Lysine	6.0	6.3	4.7	4.8	5.9	6.4	6.0	6.0	7.3	7.2	5.9	5.9
Methionine	2.2	2.3	2.2	2.1	2.0	1.9	2.0	1.9	2.3	2.2	2.3	2.2
Phenylalanine	2.2	2.4	2.1	2.0	2.0	2.1	2.2	2.2	2.3	2.3	2.3	2.1
Threonine	3.7	3.8	3.3	4.0	3.1	3.4	2.7	3.1	3.2	3.2	3.0	2.9
Valine	3.4	3.6	3.0	2.9	3.2	3.5	3.2	3.4	3.4	3.7	3.2	3.3
Sum IAA	33.9	35.2	30.2	30.3	32.6	34.1	31.6	32.4	33.2	33.8	30.5	30.1
DAA^c^												
Alanine	6.8	6.5	6.5	6.8	5.3	5.0	6.1	6.0	5.3	5.3	5.5	5.7
Asparagine	7.6	7.3	6.4	6.7	5.8	6.2	6.4	6.4	7.2	7.2	6.5	6.5
Glutamine	11.4	11.1	10.2	10.3	9.3	9.2	9.7	10.0	11.2	11.0	10.0	10.2
Glycine	10.6	9.6	12.8	12.7	6.9	5.0	8.8	8.9	5.6	5.7	7.8	7.8
Hydroxyproline	2.7	2.1	3.4	3.3	1.5	0.65	2.0	2.0	0.99	0.91	1.8	1.8
Proline	4.9	4.8	5.7	6.3	3.4	3.0	4.2	4.5	3.2	3.5	4.2	4.2
Serine	4.1	3.9	3.9	4.0	3.3	3.0	3.7	3.8	3.4	3.2	3.5	3.3
Tyrosine	1.6	1.8	1.4	1.1	1.4	1.8	1.6	1.7	1.9	1.9	1.8	1.8
Sum DAA	49.7	47.1	50.3	51.2	36.9	33.9	42.5	43.3	38.8	38.7	34.6	41.3
Sum protein AA	83.6	82.3	80.5	81.5	69.5	68.0	74.1	75.7	72.0	72.5	71.6	71.4

*^a^Allowed replicate variation: RSD <6% for 2/3 of amino acids.*

*^b^Indispensable amino acids.*

*^c^Dispensable amino acids*.

The molecular weight distribution (MWD) of the peptides (Table 5) showed that the hydrolysates mainly contained peptides <4 kDa, equivalent to peptides <20 amino acid units (40). Peptides of <25 amino acids may have bioactive properties (41), which adds to the nutritional value of the hydrolysates. Especially hydrolysates based on mackerel and herring contained high levels of molecules <0.2 kDa, mostly being small dipeptides and free amino acids. The enzymes were dosed to obtain comparable hydrolysis process for all raw materials, and the discrepancy in MWD may be explained with higher levels of free amino acids in the mackerel- and herring-based substrates. In addition, the effect of postmortem changes and presence of endogenous enzymes may add to the hydrolysis process, as the postmortem metabolomic activity of pelagic species is known to be high (42, 43). Furthermore, slightly higher levels of molecules <0.2 kDa were observed in Food Pro PNL-hydrolysates compared with those based on Bromelain. As the applied enzymes were declared endopeptidases, the amount of free amino acids should reflect upon the levels in the raw materials, and not be affected by hydrolysis. However, the increase in molecules <0.2 kDa has also been observed in previous research and indicate that Food Pro PNL has some exopeptidase activity (14).

**Table 5:** Molecular weight distribution (wt %) of hydrolysates based on salmon (S), mackerel, and herring (H) backbones (B) and heads (H) using the enzymes Food Pro PNL (-FP) and Bromelain (-B).

	**Salmon**				**Mackerel**				**Herring**			
	**Backbone**		**Head**		**Backbone**		**Head**		**Backbone**		**Head**	
**MW (kDa)**	**SB-FP**	**SB-B**	**SH-FP**	**SH-B**	**MB-FP**	**MB-B**	**MH-FP**	**MH-B**	**HB-FP**	**HB-B**	**HH-FP**	**HH-B**
>20	<0.01	0.1	0.1	<0.01	0.2	0.1	0.1	<0.01	<0.01	0.1	<0.01	<0.01
20–15	0.1	<0.01	0.1	<0.01	0.1	<0.01	0.5	<0.01	<0.01	<0.01	<0.01	<0.01
15–10	0.4	<0.01	0.5	0.1	0.3	0.1	2.3	<0.01	0.1	<0.01	<0.01	0.1
10–8	0.9	<0.01	1.1	0.2	0.6	0.1	2.7	<0.01	0.2	0.1	0.1	0.1
8–6	3.1	0.3	4.0	1.1	2.1	0.4	4.9	0.2	0.7	0.4	0.3	0.7
6–4	7.6	1.9	93	4.8	6.3	1.9	9.3	1.2	2.9	1.9	1.7	2.8
4–2	19.4	12.8	22.9	21.4	17.0	12.0	16.0	7.6	10.5	10.4	10.1	13.9
2–1	18.4	24.3	19.3	26.7	17.2	23.0	14.6	18.0	17.2	20.8	21.2	22.5
1–0.5	15.3	23.5	13.7	18.5	15.0	22.6	11.7	23.3	19.9	23.1	24.4	19.5
0.5–0.2	15.1	19.2	10.5	10.6	12.9	15.6	10.9	21.3	19.5	18.2	19.5	13.1
<0.2	19.7	17.9	18.5	16.3	28.3	24.1	27.1	28.3	29.1	25.1	22.5	27.3

### Sensory Properties

**Figure 1:**
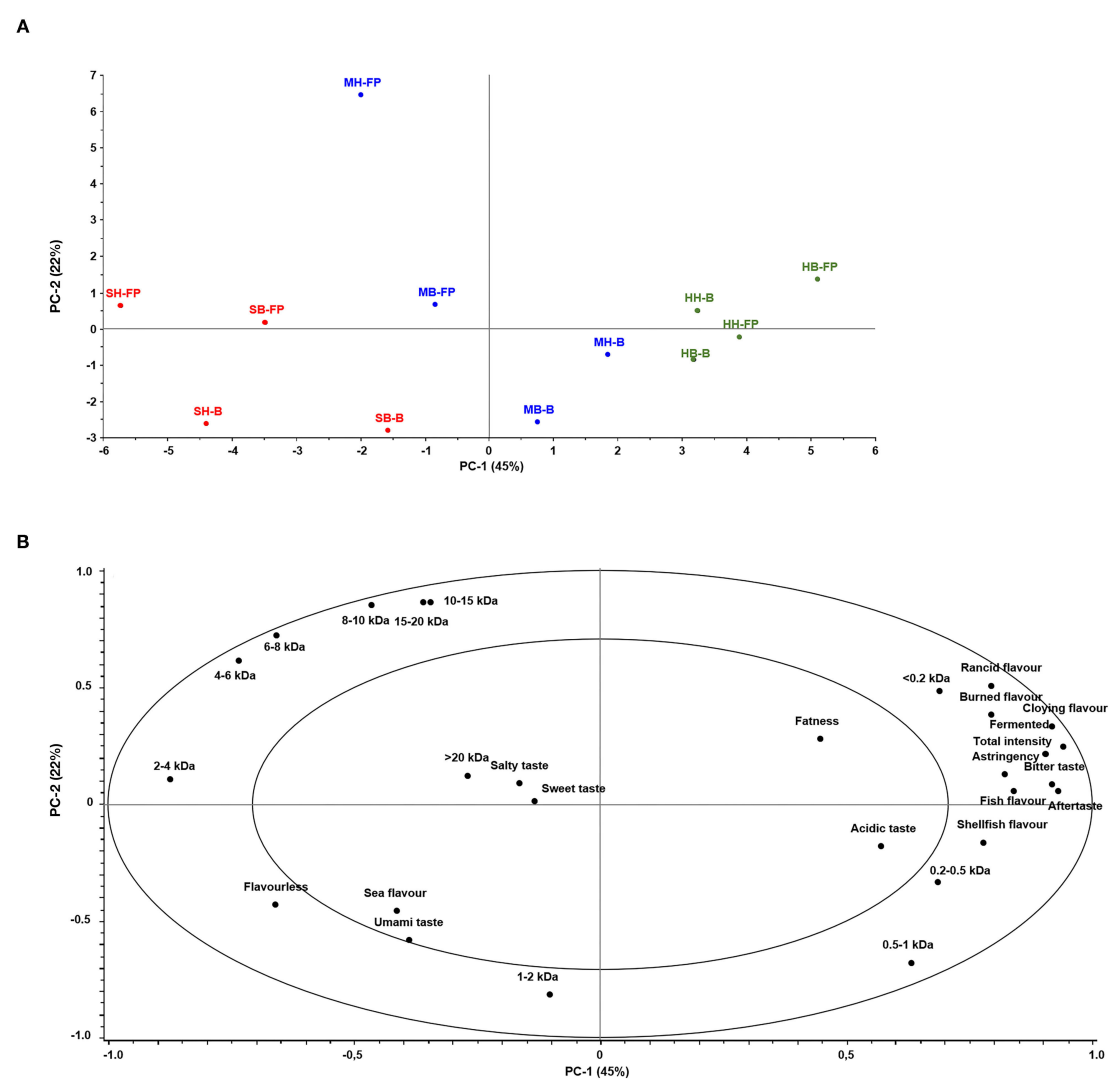
Principal component analysis score plot of hydrolysates based on salmon (S; red), mackerel (M; blue), and herring (H; green) **(A)** and loading plot of sensory properties and molecular weight distribution **(B)**. Sample coding: H, heads; B, Backbones; -FP, Food Pro PNL; -B, Bromelain.

The sensory properties of fish-based hydrolysates are of utmost importance to evaluate their potential use in products intended for human consumption. Generic descriptive analysis was performed on the hydrolysates to compare the effect of fish species, side stream fractions, and choice of enzyme on various sensory attributes (Table 1). Principal component analysis (PCA) was used to evaluate the association between sensory properties and MWD of the different hydrolysates. The first and the second principal components (PCs) explained 45 and 22% of the variance, respectively. The third and fourth PCs explained 8 and 5%, respectively (not shown). The PCA score plot (Figure 1A) shows that the hydrolysates were mostly separated based on specie; hydrolysates from salmon and herring were negatively correlated on PC1. PC2 mostly explains the effect of enzymes on the product variation, which has been found in several previous studies (8, 14). However, the higher variation explanation of the former PC illustrates the considerable raw material effect.

Based on the PCA loading plot (Figure 1B), it was evident that hydrolysates based on salmon were associated with larger peptides (>2 kDa), low flavor intensity, and pleasant flavors, such as sea flavor and umami taste. All herring hydrolysates were associated with small peptides (<1 kDa) and high sensory intensity of most of the tested attributes, including total intensity of flavor, bitter, rancid, burned, cloying, fermented fish, aftertaste, shellfish, and acidic tastes and flavors. It has been suggested that oxidation products play a part in the development of bitter tastes (12) and the positive association between rancid flavor and bitter taste with herring hydrolysates indicates that some oxidation has occurred during, or prior, to hydrolysis of the herring raw material. It was, however, found to have comparable or lower lipid contents compared with the other raw materials (Table 2), possibly indicating higher prooxidative activity from blood components of herring. A study on oxidation effects of hemolysate from trout, herring, and mackerel found the latter two to have more rapid deterioration effects on cod compared with the former (44). In the current study other factors may have added to the oxidation in herring, such as amount of remnant blood in the raw material, other blood components, and potential postmortem changes.

The mackerel hydrolysates were mostly in the center of the plot and associated with the lesser explained (<50%) attributes, such as acidic, salty, sweet, sea flavor and fatness, and molecules <20 kDa. Furthermore, the mackerel hydrolysates based on Food Pro PNL were mostly similar to the salmon hydrolysates, whereas the mackerel hydrolysates based on Bromelain were more similar to herring hydrolysates (Figure 1A). This indicates that Bromelain produces hydrolysates of higher taste-intensity compared with Food Pro PNL in mackerel raw material.

The mean intensity values for the individual sensory attributes showed that, except for sweet taste and shellfish and marine flavors, all tested attributes varied significantly (*p* <0.05) between the products (Table 6). In general, the products were taste and flavor intense, with total flavor intensity scores between 4.7 and 7. Hydrolysates based on herring were particularly flavor intense, bitter, and with high scores for fermented fish flavor, followed by mackerel and salmon hydrolysates. Fermented fish flavor is ascribed to “bad or stale fish” (Table 1), indicating that the herring hydrolysates were the most unpalatable of the assessed products. The hydrolysates were filtered post-hydrolysis to remove residual fat from the products and the overall intensity of rancid flavor was low. However, higher levels in the herring hydrolysates suggests some lipid oxidation in the raw material before or during the hydrolysis process, as discussed above. The unpalatability of hydrolysates from pelagic species may also be ascribed to their relatively high ratio of dark muscle and the accompanying prooxidative components such as copper and iron, compared with salmon (45). Mackerel and herring (and other pelagic species) are exempt from the regulatory requirement for bleeding upon loading (46). This may result in more blood in the side stream materials, affecting both the molecular composition and sensory properties of the resulting hydrolysate. Another possible explanation may be the difference in fatty acid composition of the raw materials (Table 2), with varying levels of fatty acids susceptible for oxidation during storage, with docosahexaenoic acid being the most susceptible (47). Although practically all lipids were removed in the downstream filtration process, there might have been oxidation products present in the final hydrolysates.

**Table 6:** Mean sensory intensity values of protein hydrolysates based on salmon (S), mackerel (M), and herring backbones (B) and heads (H) using Food Pro PNL (-FB) and Bromelain (-B).

	**Salmon**				**Mackerel**				**Herring**				
	**Backbone**		**Head**		**Backbone**		**Head**		**Backbone**		**Head**		
	**SB-FB**	**SB-B**	**SH-FP**	**SH-B**	**MB-FP**	**MB-B**	**MH-FP**	**MH-B**	**HB-FP**	**HB-B**	**HH-FP**	**HH-B**	***p*-value**
Total intensity of flavor	5.25^cde^	5.59^bcde^	4.96^de^	4.72^e^	6.23^abcd^	6.33^abc^	5.94^abcde^	5.75^abcde^	7.01^a^	6.38^abc^	6.39^abc^	6.59^ab^	<0.001
Sweet taste	2.94	2.48	2.47	2.60	2.53	2.95	2.70	2.67	2.57	2.44	2.57	2.57	0.559
Salty taste	2.67^ab^	2.24^b^	3.56^a^	3.11^ab^	2.91^ab^	3.12^ab^	2.88^ab^	2.98^ab^	2.67^ab^	2.70^ab^	3.23^ab^	3.19^ab^	0.01
Acidic taste	2.26^b^	2.18^b^	2.04^b^	2.14^b^	2.64^ab^	3.09^a^	2.19^b^	2.17^b^	2.69^ab^	2.59^ab^	2.42^b^	2.50^ab^	<0.001
Bitter taste	4.36^bc^	4.74^abc^	3.25^c^	3.91^bc^	4.71^abc^	4.66^abc^	4.72^abc^	5.02^ab^	6.05^a^	5.98^a^	5.23^ab^	5.13^ab^	<0.001
Umami taste	3.91^ab^	3.59^ab^	3.91^ab^	3.71^ab^	3.69^ab^	4.58^a^	2.87^b^	3.21^b^	3.02^b^	3.28^ab^	3.33^ab^	3.62^ab^	0.005
Fish flavor	3.20^b^	3.17^b^	3.31^b^	4.22^ab^	4.64^ab^	5.17^a^	4.32^ab^	4.49^ab^	5.30^a^	5.29^a^	5.45^a^	5.56^a^	<0.001
Marine flavor	1.63	1.45	1.61	1.99	2.03	2.06	1.35	1.49	1.34	1.62	1.34	1.32	0.05
Shellfish flavor	2.77	2.08	2.36	2.34	2.99	3.60	2.32	3.16	3.11	3.64	3.48	3.44	0.06
Burned flavor	1.68^a^	1.51^a^	1.22^a^	1.17^a^	1.40^a^	1.57^a^	2.06^a^	1.50^a^	2.53^a^	2.11^a^	2.56^a^	2.09^a^	0.009
Rancid flavor	1.10^c^	1.11^c^	1.18^c^	1.12^c^	1.62^bc^	1.15^c^	2.49^abc^	2.03^abc^	3.36^a^	2.14^abc^	2.88^a^	2.66^abc^	<0.001
Fermented fish flavor	1.32^c^	1.66^c^	1.29^c^	1.55^c^	3.23^abc^	2.91^bc^	3.14^abc^	3.38^abc^	5.21^a^	4.34^ab^	4.71^ab^	4.85^ab^	<0.001
Flavorless flavor	2.78^abc^	4.30^a^	3.27^abc^	3.98^ab^	3.15^abc^	3.07^abc^	2.97^abc^	2.69^abc^	2.29^c^	2.98^abc^	2.45^bc^	2.15^c^	0.001
Cloying flavor	1.58^d^	2.02^d^	1.73^d^	1.92^d^	3.34^cd^	3.24^cd^	4.24^bc^	4.46^abc^	6.31^a^	4.74^abc^	5.48^ab^	5.51^ab^	<0.001
Astringency	3.58^abc^	4.00^abc^	2.91^c^	2.96^c^	3.84^abc^	3.52^abc^	3.63^abc^	3.38^bc^	4.75^a^	4.37^ab^	4.14^abc^	4.07^abc^	<0.001
Fatness	2.72^a^	2.90^a^	2.74^a^	2.81^a^	2.87^a^	3.06^a^	3.24^a^	3.31^a^	2.88^a^	2.74^a^	3.16^a^	3.45^a^	0.008
Aftertaste	4.33^c^	4.72^bc^	4.32^c^	4.21^c^	5.31^abc^	5.71^ab^	4.89^abc^	5.10^abc^	6.00^a^	5.61^ab^	5.64^ab^	5.66^ab^	<0.001

*Different letters indicate statistical difference (p <0.05) among the hydrolysates by two-way ANOVA and Tukey's multiple comparison test*.

**Figure 2:**
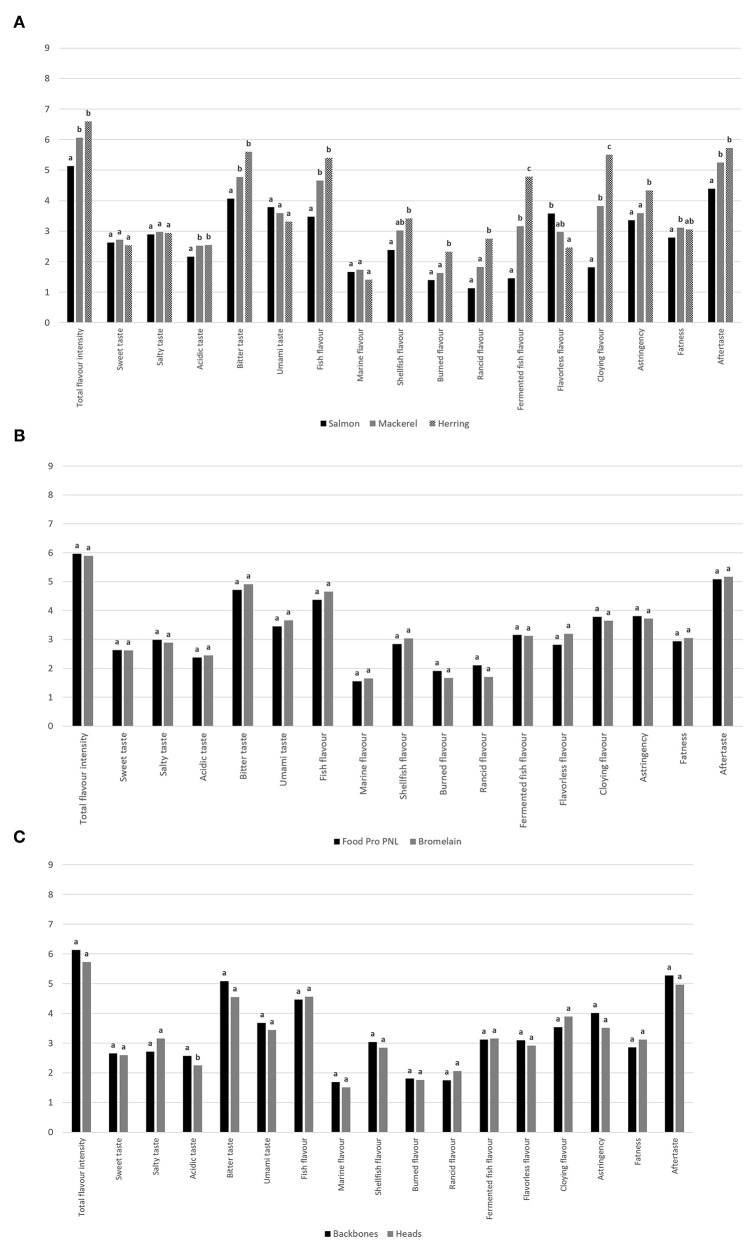
Mean intensity of sensory attributes of protein hydrolysates based on **(A)** salmon, mackerel, and herring, **(B)** Food Pro PNL and Bromelain, and **(C)** heads and backbones. Different letters indicate statistical difference within each variable (*p* < 0.05) based on mixed model ANOVA and Tukey's multiple comparison test.

The individual effects of fish species, side stream fraction, and enzyme on the hydrolysates' sensory attributes were evaluated using mixed model ANOVA (Figure 2). Products based on herring and mackerel were significantly more flavor intense and had significantly higher intensity of the attributes, such as acidic, bitter and fish, and with a stronger aftertaste compared with hydrolysates based on salmon (Figure 2A). Although not significant at *p* < 0.05, the levels were higher for herring-hydrolysates compared with mackerel for the above-mentioned attributes. Further, hydrolysates based on herring were significantly more cloying and had a higher intensity of fermented fish flavor compared with both salmon and mackerel. Flavourlessness was the only attribute where salmon had the highest intensity, and this suggests that salmon raw material was the most applicable substrate for production of taste-acceptable and palatable hydrolysates.

No significant effects of neither enzyme (Figure 2B) nor fraction (Figure 2C) were observed, with the exception of acidic taste being higher in the backbone hydrolysates. The findings were in agreement with previous studies on salmon and mackerel raw materials (10), and indicate similar levels of peptides and molecules influencing sensory attributes in head- and backbone hydrolysates, both substrates being rich in muscle protein and connective tissues. Choice of enzyme is an important factor in the production of protein hydrolysates, as this will influence both processing costs (21) and product sensory properties (14, 19, 20, 30). Bromelain has been found to produce significantly higher bitter taste intensity compared with Food Pro PNL in fish hydrolysates (14); however, in the present study only small and insignificant differences between the two proteases were observed (Figure 2B). Further, Bromelain has been found to produce hydrolysates of less bitter taste compared with Alcalase in soy protein isolates (20), reflecting that both raw material composition and processing conditions are important for the development of bitter taste. The findings from this work indicate that choice of fish raw material and post-harvest handling may be the most important determinator for bitter and unpalatable taste development, where herring-based products were the least palatable compared with mackerel and salmon. Although fish-based protein hydrolysates are good sources of protein and IAA, they must be palatable to achieve consumer acceptance if they are to be used as food ingredients.

## Conclusions

Hydrolysates based on herring were the most flavor-intense and bitter, followed by mackerel and salmon. Only small and insignificant effects of fraction (i.e., head vs. backbone) and choice of enzyme (i.e., Bromelain vs. Food Pro PNL) were observed on the tested sensory properties. All hydrolysates contained high levels of connective tissue amino acids glycine, proline, and a well-balanced amino acid composition. Hydrolysates based on heads were richer in ash compared with backbone-based hydrolysates, suggesting more bone material in the heads. The study indicates that salmon side stream materials, both heads and backbones, are more suited toward human consumption, compared with herring and mackerel. Additional raw material preparation and more focus on post-harvest handling to reduce potential prooxidative components should be investigated to produce palatable products from pelagic species.

## Data Availability Statement

The original contributions presented in the study are included in the article/supplementary material, further inquiries can be directed to the corresponding author/s.

## Author Contributions

TA, SS, and KK: conceptualization. TA, SS, BV, MC, JA, and KK: methodology, validation, visualization, and writing—review and editing. TA and MC: software. SS, BV, MC, and JA: formal analysis and investigation. KK: resources. TA: data curation and writing—original draft preparation. TA and KK: supervision. KK: project administration and funding acquisition. All authors have read and agreed to the published version of the manuscript.

## Funding

The work was funded by the EU Commission through the BBI-JU H2020 Project AQUABIOPRO-FIT Aquaculture and agriculture biomass side stream proteins and bioactives for feed, fitness and health promoting nutritional supplements (grant number 790956). The Research Council of Norway is acknowledged for the support to the Aquafeed Technology Centre, ATC (project number 245883/F50).

## Conflict of Interest

The authors declare that the research was conducted in the absence of any commercial or financial relationships that could be construed as a potential conflict of interest.

## Publisher's Note

All claims expressed in this article are solely those of the authors and do not necessarily represent those of their affiliated organizations, or those of the publisher, the editors and the reviewers. Any product that may be evaluated in this article, or claim that may be made by its manufacturer, is not guaranteed or endorsed by the publisher.
